# Generation of murine tumor cell lines deficient in MHC molecule surface expression using the CRISPR/Cas9 system

**DOI:** 10.1371/journal.pone.0174077

**Published:** 2017-03-16

**Authors:** Krishna Das, David Eisel, Clarissa Lenkl, Ashish Goyal, Sven Diederichs, Elke Dickes, Wolfram Osen, Stefan B. Eichmüller

**Affiliations:** 1 GMP & T Cell Therapy Unit, German Cancer Research Center (DKFZ), Heidelberg, Germany; 2 Division of RNA Biology and Cancer, German Cancer Research Center (DKFZ), Heidelberg, Germany; 3 Division of Cancer Research, Dept. of Thoracic Surgery, Medical Center - University of Freiburg, Faculty of Medicine, University of Freiburg & German Cancer Consortium (DKTK), Freiburg, Germany; Osaka University, JAPAN

## Abstract

In this study, the CRISPR/Cas9 technology was used to establish murine tumor cell lines, devoid of MHC I or MHC II surface expression, respectively. The melanoma cell line B16F10 and the murine breast cancer cell line EO-771, the latter stably expressing the tumor antigen NY-BR-1 (EO-NY), were transfected with an expression plasmid encoding a β_2_m-specific single guide (sg)RNA and Cas9. The resulting MHC I negative cells were sorted by flow cytometry to obtain single cell clones, and loss of susceptibility of peptide pulsed MHC I negative clones to peptide-specific CTL recognition was determined by IFNγ ELISpot assay. The β_2_m knockout (KO) clones did not give rise to tumors in syngeneic mice (C57BL/6N), unless NK cells were depleted, suggesting that outgrowth of the β_2_m KO cell lines was controlled by NK cells. Using sgRNAs targeting the β-chain encoding locus of the IA^b^ molecule we also generated several B16F10 MHC II KO clones. Peptide loaded B16F10 MHC II KO cells were insusceptible to recognition by OT-II cells and tumor growth was unaltered compared to parental B16F10 cells. Thus, in our hands the CRISPR/Cas9 system has proven to be an efficient straight forward strategy for the generation of MHC knockout cell lines. Such cell lines could serve as parental cells for co-transfection of compatible HLA alleles together with human tumor antigens of interest, thereby facilitating the generation of HLA matched transplantable tumor models, e.g. in HLAtg mouse strains of the newer generation, lacking cell surface expression of endogenous H2 molecules. In addition, our tumor cell lines established might offer a useful tool to investigate tumor reactive T cell responses that function independently from MHC molecule surface expression by the tumor.

## Introduction

Targeted genome editing, i. e. introduction of artificial sequence changes at targeted sites within the genome, has become a standard procedure in molecular biology. Recently, the bacterial “Clustered Regularly Interspaced Short Palindromic Repeats/CRISPR associated nuclease 9” (CRISPR/Cas9) system has evolved as a powerful platform for targeted genome editing. Originally, the CRISPR/Cas9 system represented a prokaryotic adaptive immune defense mechanism protecting bacteria from invasion by foreign genomes, based on the integration of foreign DNA sequences as so called protospacers into the host chromosome, separated by endogenous CRISPR sequences [1, 2]. Meanwhile, the CRISPR/Cas9 system has been adopted for routine targeted genome editing based on induction of sequence-specific DNA double strand breaks (DSB) [3]. In fact, this technique has been successfully applied to genetically modify bacteria [4] or mammalian cell lines [5] and was furthermore used for generation of various knockout mouse strains [6].

T lymphocytes (T cells) of the adaptive immune system recognize short peptides derived from endogenous cellular proteins or from antigens of exogenous origin presented by MHC I and MHC II molecules on the cell surface. Whereas MHC class I (MHC I) molecules are expressed by any nucleated cell, expression of MHC class II molecules (MHC II) is restricted mainly to antigen presenting cells such as macrophages, B cells or dendritic cells [7]. The repertoire of MHC I restricted peptides is permanently scanned by CD8^+^ cytotoxic T cells (CTLs) that, when activated upon recognition of a foreign peptide, will eventually eradicate the epitope presenting target cell [8]. Conversely, natural killer (NK) cells of the innate immune system preferentially attack target cells that are devoid of MHC I surface expression, but display activating NK cell receptors on their surface [9]. Since formation of stable MHC I/peptide complex is critically dependent on the association with β_2_m (light chain), defective β_2_m expression will abrogate stable MHC I cell surface expression [10]. In analogy to MHC I restricted epitope presentation, peptides presented by MHC II molecules are screened by CD4^+^ T cells which become activated upon recognition of a foreign peptide. As functional MHC II molecules consist of α:β heterodimers, inactivation or deletion of either peptide chain (α or β) will prevent their cell surface expression.

In our study presented here, we utilized the CRISPR/Cas9 system to knock out the β_2_m gene in murine tumor cell lines resulting in deficient MHC molecule expression on their surface. Similarly, we used the CRISPR/Cas9 technology to generate MHC II negative B16F10 cells by targeting the IA^b^ β chain encoding locus in this cell line. Our results show that the CRISPR/Cas9 system represents an efficient straight forward strategy for the generation of stable murine MHC knockout tumor cell lines. We conclude that the MHCI/II deficient tumor cell lines generated by the CRISPR/Cas9 technology might serve as parental lines for the establishment of MHC compatible, transplantable tumor models in HLA-transgenic mouse strains devoid of endogenous MHC molecule expression. Moreover, our knockout cell lines might offer a useful tool to investigate tumor reactive T cell responses that function independently from MHC molecule surface expression by the tumor.

## Material and methods

### Generation of single guide RNA encoding plasmids

Single guide (sg)RNAs targeting exon 1 of the murine β_2_m gene or the IA^b^ beta chain gene were designed using the online sgRNA design tool available at https://crispr.mit.edu/. In order to maximize specificity, guide sequences with high scores for on-target activity and at least 3 base pair mismatches to any predicted off-targets in the genome were selected. The corresponding sense and antisense DNA oligomers (obtained from Sigma) are shown in S1 Table. Annealed oligomers were cloned downstream of the U6 promoter of PX458 (Addgene, Plasmid 48138, Middlesex, UK) as described earlier (Ran, 2013). A plasmid map of PX458 is depicted in S1 Fig.

### Cell lines

The murine C57BL/6 derived melanoma cell line B16F10 used to knock out expression of β_2_m or IA^b^ were kindly provided by Dirk Schadendorf or purchased from ATCC, respectively. The mammary adenocarcinoma cell line EO-771 was purchased from TEBU-Bio (Offenbach, Germany). All cell lines were propagated in RPMI 1640 supplemented with Glutamax, 10% FCS, and 1% penicillin-streptomycin. The EO-771-derived transfectant clone EO-NY expressing the human breast cancer associated tumor antigen NY-BR-1 [11] established in our lab (Das et al. unpublished) was cultured in the same medium containing 0.4 mg/ml zeocin (Invitrogen). The TRP-2-specific CTL line recognizing the H2-K^b^-restricted epitope SVYDFFVWL (aa 180–188) [12] was expanded by periodical *in vitro* re-stimulation in CTL medium and has been described elsewhere [13]. T cell lines recognizing the IA^b^-restricted ovalbumin-specific epitope ISQAVHAAHAEINEAGR (aa 323–339) were generated by isolation of CD4^+^ T cells from OT-II mice using a CD4 negative selection Kit (Miltenyi), followed and activation with CD3/CD28 Dynabeads (Thermo Fisher Scientific) for seven days. After removal of the magnetic beads, CD4^+^ T cells were further expanded for two weeks by *in vitro* restimulation using C57BL/6 splenocytes and 2 μg/ml of the relevant peptide.

### IFNγ ELIspot assays

IFNγ ELISpot assays were performed using Multiscreen ELISpot plates (Millipore, Schwalbach, Germany) coated with 1 μg/ml goat anti-mouse IFNγ capture antibody (Becton Dickinson) for 1 to 2 h at 37°C or overnight at 4°C. After blocking, graded numbers of TRP-2- or ovalbumin-specific T cells were added to 5 x 10^4^ target cells in a total volume of 200 μl per well and cells were co-cultured for 16–18 h. In some cases, peptide loaded target cells (1 μg/ml; 45 min, 37°C) were used after 3–5 centrifugation rounds to wash off unbound peptides. Next day, cells were incubated with 2 μg/ml biotinylated rat anti-mouse IFNγ antibody (Becton Dickinson) followed by incubation with avidin-conjugated alkaline phosphatase (Becton Dickinson). IFNγ-specific spots were developed by addition of BCIP/NBT (Sigma) and reaction was stopped with distilled water. Spots were counted using an ELISpot reader (AID, Strassberg, Germany). In some experiments, B16F10 cells were pretreated with 20 U/ml IFNγ (eBioscience, Frankfurt, Germany) for 48 h to induce IA^b^ expression, prior to the assay.

### IFNγ ELISA with NK cells

NK cells were isolated from splenocytes of C57BL/6 mice using NK Cell Isolation Kit II, (Miltenyi Biotec) and activated for seven days with 1,700 IU/ml IL-2 (Miltenyi Biotec). Activated NK cells (5 x 10^4^) were incubated with 2.5 x 10^5^ target cells for 8 hours in a total volume of 200 μl. Supernatant was collected and IFNγ secreted by NK cells was quantified using the Mouse IFN gamma ELISA Ready-SET-Go! Kit (Affymetrix, eBioscience), according to manufacturer’s instructions.

### Peptides

Peptides were synthetized by Fmoc chemistry [14, 15] using a fully automated multiple synthesizer Syro II (MultiSyn Tech, Germany), followed by HPLC purification on a Kromasil 100–10C 10 μm 120A reverse phase column (20 x 150 mm). Eluted peptides were analyzed by HPLC and MS (Thermo Finnigan LCQ).

### Immunofluorescence staining and flow cytometry

Immunofluorescence staining of murine tumor cell lines was performed using culture supernatant of following murine hybridomas, kindly provided by Günter Hämmerling (DKFZ): S19.4 (anti-β_2_m^b^) [16], E3-25 (anti-H2-K^b^) [17], B22.249 (anti-H2-D^b^) [18]. Staining of IA^b^ molecules was performed using mononclonal antibody AF6-120 coupled to PE (BD) or to Alexa Fluor 647 (Biolegend, Fell, Germany), respectively. As controls, culture supernatants of hybridomas secreting isotype matched antibodies against irrelevant epitopes were included. Cells (2 x 10^5^) were incubated with 100 μl hybridoma supernatant for 1 hour at 4°C, followed by two washing steps and subsequent incubation with FITC-conjugated goat anti-mouse IgG (BD) as second antibody. Alternatively, cells were incubated with fluorochrome conjugated IA^b^-specific antibody AF6-120 diluted in PBS containing 3% FCS. Finally, after staining with 7-AAD for live/dead discrimination, cells were analyzed with a FACSCalibur cytometer and data were evaluated with FlowJo software.

### Animal experiments

Animal experiments were approved by the internal ethics committee of the German Cancer Research Center and by the District Government in Karlsruhe, Germany. C57BL/6 mice (Charles River, Sulzfeld) were injected s.c. into the flank with 2 x 10^5^ B16F10 cells or with the same number of EO-NY transfectants. Tumor growth was monitored twice per week and mice were killed after 17 days or when the tumor area had reached a size of 225 mm^2^. Mice were sacrificed by gradual CO_2_ exposition. For NK cell depletion, 100 μg NK1.1 specific monoclonal antibody clone PK136, or isotype control clone C1.18.4 (both BioXcell, West Lebanon, NH) were administered intraperitoneally 2 days prior to the day of tumor injection. To maintain NK cell depletion, antibody injection was repeated on the day of tumor injection and on days 7 and 13 after tumor inoculation.

### Transfection and fluorescence-activated cell sorting

One day before transfection, 2 x 10^5^ B16F10 cells or 2.5 x 10^5^ EO-NY cells, respectively, were seeded per well of a 6-well plate. Next day, 0.8 μg of guide RNA encoding plasmid was transfected applying the Effectene Transfection Reagent (Qiagen, Hilden, Germany), according to the manufacturer’s instructions. The proportion of transfected cells expressing EGFP was determined by FACS two to three days after transfection or at time points stated in the text. To generate stable knockout clones, EGFP expressing transfectants were sorted by FACS two to three days after transfection and expanded *in vitro* for 7 days. Cells underwent immunofluorescence staining with H2D^b^–specific antibody B22.239 and H2D^b^ negative cells were cloned by limiting dilution (B16F10 β_2_m KO cells) or by FACS guided single cell sorting (EO-NY β_2_m KO cells). EGFP expressing B16F10 IA^b^ KO cells were treated with 20 U/ml IFNγ for 72 h, prior to IA^b^-specific immunofluorescence staining. Subsequently, IA^b^ negative cells were cloned by FACS guided single cell sorting.

### DNA sequencing

CRISPR/Cas9 induced mutations were analyzed on genomic DNA isolated from the generated KO clones as well as from the parental cell lines using the QIAamp DNA Blood Mini Kit (Qiagen). The sequences of interest containing the predicted cutting sites of the Cas9 nuclease were amplified by PCR using 0.5 μM of each primer depicted in S2 Table (Sigma), 0.2 mM dNTP Mix (VWR), 2.5 mM MgCl_2_ (Thermo Fisher Scientific), 1.25 U Taq polymerase (Thermo Fisher Scientific), and 160 ng genomic DNA resolved in Taq Buffer (Thermo Fisher Scientific). The PCR products were purified using the QIAquick PCR Purification Kit (Qiagen) and subsequently integrated into the TOPO vector using the TOPO TA Cloning Kit for sequencing (Thermo Fisher Scientific). After heat shock transformation of One Shot competent TOP10 bacteria (Thermo Fisher Scientific), cells were plated on ampicillin containing LB-plates and incubated overnight. Next day, picked clones were expanded overnight in LB medium supplemented with 50 μg/ml ampicillin (37°C; 200 rpm). Plasmids were isolated using QIAprep Spin Miniprep Kit (Qiagen) according to the manufacturer’s instructions and sent to GATC Biotech (Cologne, Germany) for sequencing. Data were analyzed using the sequence ApE-A plasmid Editor (M. Wayne Davis).

## Results

### Generation and characterization of β_2_m-specific guide RNAs

In order to generate MHC I negative murine tumor cell lines we targeted the β_2_m gene, since association of β_2_m with the MHC I heavy chain is essential for stable complex formation and hence for efficient epitope presentation on the cell surface [10]. Based on the CRISPR Design Tool (https://crispr.mit.edu/), two DNA oligomers whose transcripts showed high on-target scores for exon 1 of the murine β_2_m gene with minimal predicted off-target activity (S1 Table) were selected. The oligomers were cloned into the Bbs1 site of vector PX458 (S1 Fig), resulting in the vectors PX458_mβ_2_m guide #1 and PX458_mβ_2_m guide #2, abbreviated PX458/#1 and PX458/#2, respectively. In pilot experiments using these constructs to transfect EO-NY cells, transfection efficiencies between 11.2% and 15.5% were achieved (not shown).

**Fig 1:**
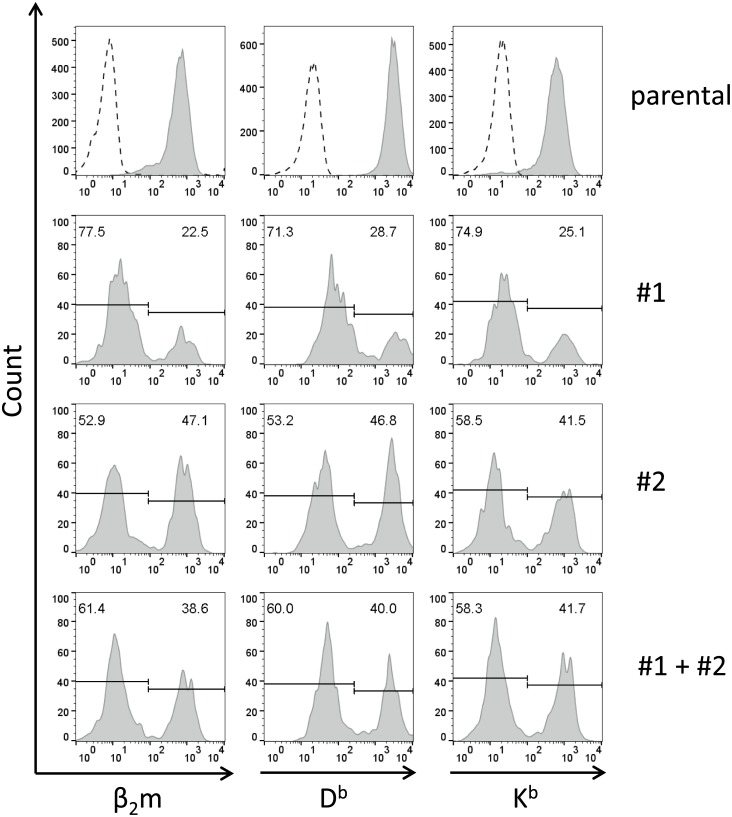
Transfection of EO-NY cells with guide#1 and guide#2 RNA encoding constructs results in outgrowth of β_2_m negative cell populations. EO-NY cells transfected with guide#1 or guide #2 constructs, respectively (middle panels), or with pooled guide#1 and guide#2 constructs (lower panel) were stained with monoclonal antibodies specific for β_2_m (left), H2-D^b^ (center) or H2-K^b^ molecules (right) and analyzed by FACS seven days after transfection. When gated on EGFP expressing transfectants, β_2_m negative subpopulations showed up in transfected bulk cultures, but not among parental EO-NY cells (upper panel). Note that the proportion β_2_m negative cells was equal to the proportion of transfectants lacking MHC I molecule expression.

Next, the two newly generated β_2_m-specific sgRNA constructs were tested for their capacity to inactivate β_2_m expression in murine tumor cell lines. Thus, plasmids PX458/#1 and PX458/#2 were transfected into EO-NY cells, and β_2_m expression on the surface of transfected cells was analyzed by flow cytometry seven days later. When gated on EGFP^+^ cells, thereby restricting the analysis to EO-NY cells efficiently transfected with the sgRNA encoding constructs, a subpopulation of β_2_m negative cells became apparent among the transfected populations that was absent among parental cells (Fig 1). This subpopulation comprised 77.5% or 52.9% of cells, upon transfection with PX458/#1 or PX458/#2, respectively. Of note, the percentages of β_2_m KO cells strictly correlated with the frequencies of cells showing negative signals when stained with H2-D^b^- or H2-K^b^–specific antibodies.

Since PX458/#1 appeared superior to PX458/#2 in its efficiency to induce β_2_m deficient EO-NY cells, and due to the fact that combined transfection of both vectors could not further enhance the proportion of β_2_m negative cells (Fig 1, lower panel), PX458/#1 was selected for subsequent experiments.

### Generation of stable MHC I negative murine tumor cell lines using the CRISPR/Cas9 system

Having demonstrated the efficiency of our sgRNA-encoding vectors in generating β_2_m deficient EO-NY cells, we thought to use this technique also for the creation of stable knockout (KO) cell lines, including further tumor entities. Thus, EO-NY cells and the murine melanoma cell line B16F10 were transfected either with the construct PX458/#1 or with the control plasmid PX458, the latter encoding a nonfunctional tracrRNA and Cas9. Three days after transfection, EGFP expressing EO-NY and B16F10 bulk cultures were sorted by FACS to isolate cells that had been efficiently transfected (not shown). The resulting transfectant lines EO-NY/PX458/#1 and B16F10/PX458/#1, abbreviated as EO-NY/#1 and B16F10/#1, respectively, together with the sorted control transfectants EO-NY/PX458 and B16F10/PX458, were analyzed by flow cytometry for loss of MHC I surface expression seven days later. We found that 76.3% of EO-NY/#1 cells and 80.8% of the B16F10/#1 cells had lost surface expression of H2-D^b^ molecules, whereas 99.8% or 100% respectively, of the control transfectants had remained positive for H2-D^b^ surface expression (S2 Fig). In order to establish β_2_m KO clones from the sorted transfectant lines, D^b^-negative EO-NY/#1 single cells were seeded into individual wells by FACS guided cell sorting. Similarly, B16F10/#1 derived EGFP-expressing transfectants were cloned by conventional limiting dilution. In addition, the sorted control transfectant lines showing unaltered H2-D^b^ surface expression were cloned either by FACS (EO-NY/PX458) or by limiting dilution (B16F10/PX458) to obtain control clones with high MHC I surface expression.

**Fig 2:**
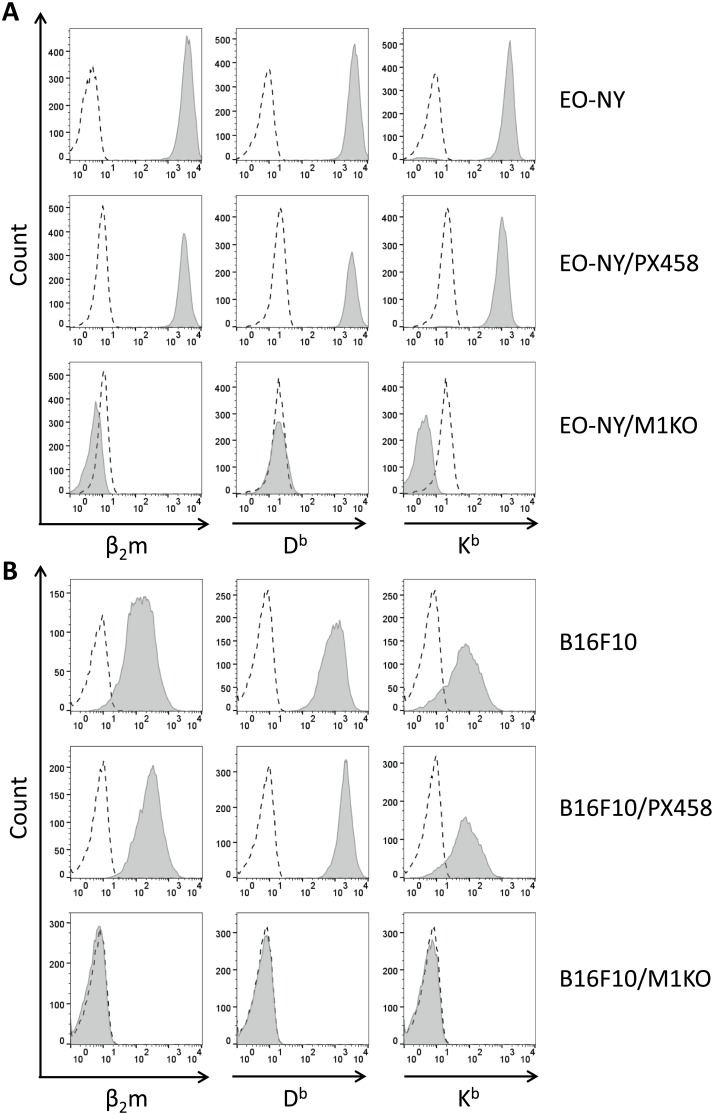
Phenotype of stable β_2_m KO clones derived from various tumor entities upon transfection with guide#1 constructs. EO-NY cells (A) or B16F10 cells (B) transfected with guide #1 constructs (A, B lower panels) or empty vector PX458 as control (A, B, middle panels) were sorted by FACS two days after transfection and cloned by limiting dilution or FACS guided single cell sorting 14 days later. Selected clones were stained with monoclonal antibodies specific for β_2_m (left), H2-D^b^ (center) or H2-K^b^ molecules (right) and analyzed by FACS. Living gate was set on 7-AAD negative cells. Guide#1 derived transfectant clones of both tumor entities showed complete loss of β_2_m as well as MHC I molecule expression (A, B, lower panel), when compared to parental cell lines (A, B, upper panel) or control transfectant clones (A, B, middle panels).

Screening of 38 EO-NY- and B16F10- derived transfectant clones lead to selection of two clones EO-NY/AD11 (S3 Fig) and B16F10/6-D3 (S4 Fig), for subsequent experiments. These clones will be referred to as EO-NY/M1KO and B16F10/M1KO, respectively, from now on throughout the manuscript. Furthermore, control clones EO-NY/F9 and B16F10/6-E8, now designated as EO-NY/PX458 and B16F10/PX458, respectively, were included. The selected clones EO-NY/M1KO and B16F10/M1KO showed complete loss of both, β_2_m and MHC I molecule expression, whereas surface expression of H2-K^b^ and—D^b^ molecules on the selected control clones (EO-NY/PX458 and B16F10/PX458) had remained unchanged, compared to the parental cell lines (Fig 2A and 2B).

### Generation of stable MHC II negative B16F10 cell lines using the CRISPR/Cas9 system

**Fig 3:**
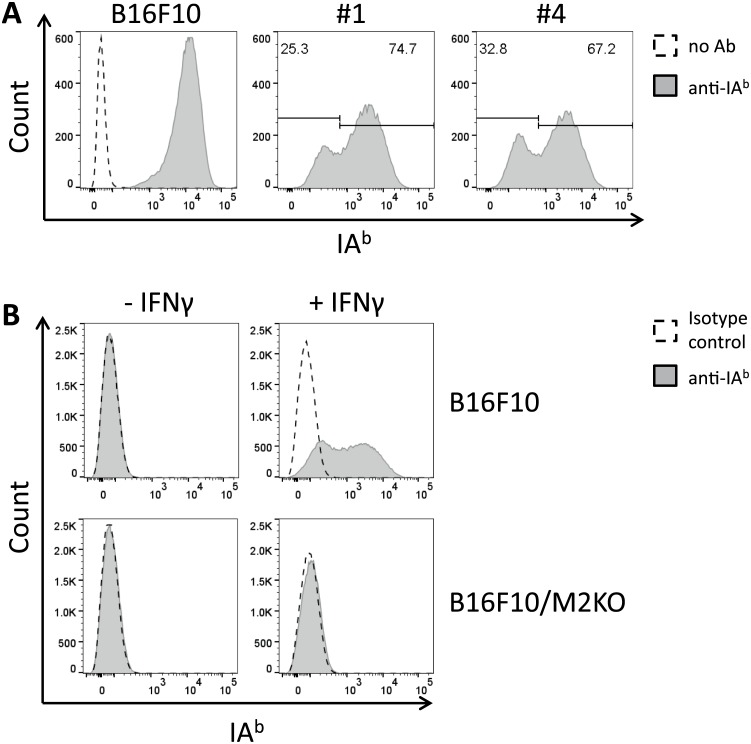
Transfection of B16F10 cells with guide #4 encoding constructs results in generation of a stable B16F10/IA^b^ KO clone. Parental B16F10 cells and B16F10 cells transfected with guide#1 or guide#4, respectively, were treated with IFNγ (20 U/ml) 9 days post transfection, followed by surface staining with IA^b^ specifc monoclonal antibody. FACS analysis performed on 7-AAD negative cells revealed a higher proportion of IA^b^ negative cells upon transfection with guide#4 (A, right histogram) compared to transfection with guide#1 (A, center). Immunofluorescence staining confirmed complete loss of IA^b^ surface expression on the selected B16F10 KO clone, even when treated with IFNγ (B, lower panel).

Next, we made use of the CRISPR/Cas9 technology to establish MHC II deficient tumor cell lines. Following the strategy described above for the generation of stable β_2_m KO clones, two oligomers encoding transcripts with high on-target activity scores for exon 1 of the IA^b^ beta chain encoding gene on chromosome 17 were selected and cloned into the Bbs1 site of PX458 (S1 Fig and S1 Table). Transfection of the resulting constructs IA_beta exon 1 guide #1 (IA_guide #1) and IA_beta exon 1 guide #4 (IA_guide #4) into B16F10 cells yielded between 48% and 60% EGFP positive cells, as determined by FACS two days after transfection (not shown). The EGFP expressing transfectants were then isolated by FACS sorting and further expanded *in vitro*. After 7 days, the cells were treated with IFNγ to stimulate IA^b^ surface expression, thus creating most stringent conditions for subsequent selection of IA^b^ KO clones. In fact, FACS analysis revealed that transfection of IA_guide#1 resulted in a subpopulation of 25.3% IA^b^-negative cells, whereas the proportion of IA^b^ negative cells induced with IA_guide#4 was 32.8% (Fig 3A). In contrast, no IA^b^ negative population was observed among parental B16F10 cells treated with IFNγ. Upon FACS guided single cell sorting 24 individual clones were established from the IA^b^ negative subpopulation of the transfected bulk culture, resulting in 23 clones with stable loss of IA^b^ surface expression (S5 Fig). Finally, clone B16F10/#4G10 was selected, from now on termed B16F10/M2KO, and expanded for subsequent experiments. FACS analysis confirmed lack of IA^b^ surface expression on IFNγ treated B16F10/M2KO cells, in contrast to parental B16F10 cells that upregulated IA^b^ expression upon IFNγ treatment (Fig 3B).

### Stable β_2_m and IA^b^ knockout clones have lost susceptibility to MHC restricted T cell recognition *in vitro*

**Fig 4:**
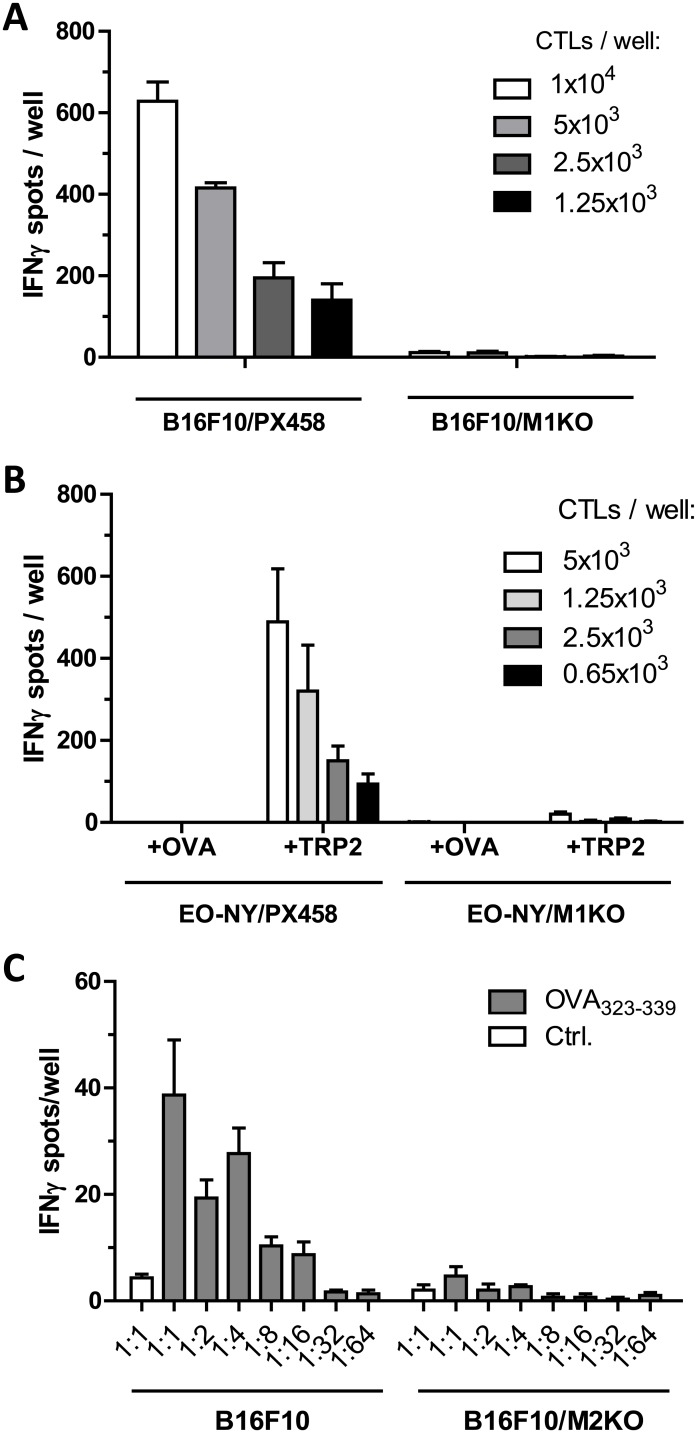
Stable β_2_m KO and IA^b^ KO clones lose susceptibility to cognate T cell recognition. B16F10/PX458 control transfectants or B16F10/M1KO cells (5 x 10^4^) were incubated overnight with graded numbers of TRP-2-specific CTLs (A). Secretion of IFNγ in response to target cell recognition was retained with B16F10/PX458 control cells but was lost with the B16F10/M1KO clone as measured by ELISpot analysis. Similarly, recognition of peptide incubated EO-NY/PX458 control cells but not of EO-NY/M1KO cells was observed upon incubation with the OVA-specific CTL line (B). Peptide loaded B16F10 cells but not B16F10/M2KO were recognized by OVA-specific OT-II cells. Target cells were treated with IFNγ (20 U/ml) prior to the assay to upregulate IA^b^ expression. Empty bars (Ctrl.), recognition of target cells loaded with IA^b^ restricted HBV core antigen control peptide 128–140 (TPPAYRPPNAPIL). Error bars represent SEM of technical triplicates (C).

In order to test, whether MHC I restricted recognition of the β_2_m KO cell clones by CD8^+^ cytotoxic T cells (CTL) would be affected, we performed IFNγ ELISpot assays with an established CTL line specific for the H2-K^b^ restricted epitope SVYDFFVWL, derived from the melanoma associated tumor antigen TRP-2 [12]. As B16F10 cells process this epitope endogenously, they could be used as direct targets for the TRP-2-specific CTLs (Fig 4A). Alternatively, EO-NY-derived β_2_m KO clones lacking endogenous expression of this tumor antigen were pulsed with the TRP-2 derived peptide prior to the ELISpot assays (Fig 4B). As shown in Fig 4A, TRP-2 expressing B16F10/PX458 cells induced IFNγ secretion by the CTL line, whereas recognition of B16F10/M1KO cells was almost completely absent. When TRP-2 negative EO-NY-derived clones were tested as targets we found that control clone EO-NY/PX458 was efficiently recognized if pulsed with the TRP-2-specific epitope, but not in the presence of an irrelevant OVA-specific K^b^-restricted CTL epitope (SIINFEKL), resulting in graded spot numbers with decreasing effector to target rations (Fig 4B). In contrast, CTL recognition of peptide pulsed EO-NY/M1KO cells was almost completely abrogated, irrespective of the peptide used for target sensitization (Fig 4B). The same recognition pattern was observed if only 100 ng/ml peptide were used for target cell sensitization (not shown).

B16F10 melanoma cells are known to show low MHC II expression under standard culture conditions but to upregulate expression of IA^b^ upon IFNγ treatment [19]. In fact, IFNγ treated parental B16F10 cells, but not B16F10/M2KO cells loaded with the IA^b^-restricted OVA-specific T cell epitope 323–339 were recognized by OT-II cells (Fig 4C), showing that IFNγ treatment was mandatory for effective T cell recognition and that the B16F10/M2KO cells had lost susceptibility to OT-II cell recognition, even when treated with IFNγ.

These results thus show that the tested β_2_m KO clones, as well as the B16F10 IA^b^ KO clone, had lost expression of the surface molecules encoded by the genes targeted through the CRISPR/Cas9 technology, resulting in lost susceptibility to MHC-restricted T cell recognition.

### B16F10 and EO-771 derived β_2_m KO clones show increased susceptibility to NK cell recognition *in vitro* and *in vivo*

**Fig 5:**
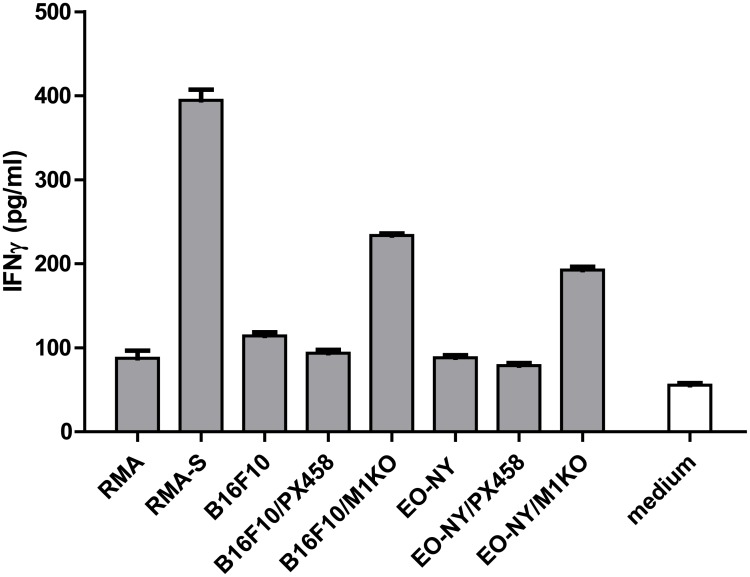
Stable β_2_m KO clones show enhanced susceptibility to NK cell recognition. Target cells (2.5 x 10^5^) were incubated with (5 x 10^4^) IL2-activated (1,700 IU/ml IL2, 7 d) NK cells for 8 h and IFNγ released by the NK cells was quantified by ELISA. B16F10/M1KO and EO-NY/M1KO clones induce enhanced IFNγ release compared to parental cells or control transfectant clones, respectively. Error bars represent SEM of technical triplicates.

Tumor cells having lost surface expression of MHC I molecules have been described as efficient targets for NK cell mediated cytolysis [9]. We thus tested our β_2_m KO tumor cell lines for recognition by activated NK cells compared to RMA-S cells, the latter representing a classical NK target [20]. Co-cultivation of clone B16F10/M1KO with NK cells induced IFNγ levels that were 2–3 fold higher compared to co-culture with parental B16F10 cells or B16F10/PX458 transfectants (Fig 5). A similar IFNγ secretion pattern was observed with the EO-771 derived transfectant clones, showing enhanced secretion of IFNγ upon co-culture with β_2_m deficient clone EO-NY/M1KO, whereas untreated EO-NY cells and control EO-NY/PX458 transfectants induced only low IFNγ secretion levels, similar to that of RMA cells. We thus conclude that the β_2_m deficient tumor cell lines generated have gained susceptibility to recognition by NK cells.

**Fig 6:**
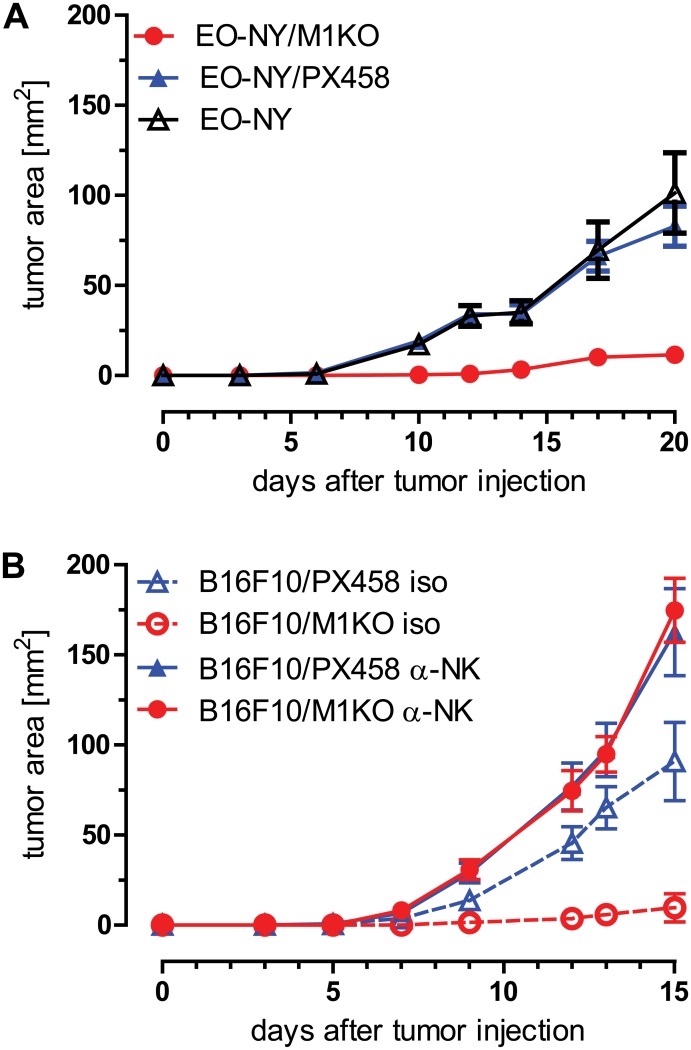
Tumor outgrowth of stable β_2_m KO clones is controlled by NK cells. C57BL/6 mice (n = 10) were injected (s.c.) with 2 x 10^5^ parental EO-NY cells or with the transfectant clones EO-NY/PX458 and EO-NY/M1KO derived thereof, showing suppressed outgrowth of EO-NY/M1KO cells (A). Similarly, 2 x 10^5^ B16F10 cells or B16F10 derived transfectant clones were injected (s.c.) into C57BL/6 mice (n = 10). NK cells were depleted by i.p. injections of monoclonal ab PK136 or isotype control on days -2, 0, 7, 13 after tumor cell application, resulting in restored tumor outgrowth (B). Error bars represent SEM within each animal collective.

We next tested the growth kinetics of the established β_2_m knockout clones *in vivo*. After s.c. injection into syngeneic C57BL/6 mice, untreated EO-NY cells and EO-NY/PX458 control transfectants showed almost superimposing tumor growth curves, reaching a tumor size of approximately 100 mm^2^ within 20 days (Fig 6A). On the contrary, the β_2_m deficient EO-NY/M1KO clone failed to grow out. A similar tumor growth pattern was observed with B16F10 derived clones: whereas control B16F10/PX458 cells readily formed tumors within two weeks, no tumor growth was observed with B16F10/M1KO cells (Fig 6B). Since B16F10/M1KO cells had proven susceptible to NK cells *in vitro* (Fig 5) we transferred these β_2_m deficient cells into C57BL/6 mice that had been depleted of NK cells and compared the tumor growth of this cell line to that of B16F10/PX458 control cells. In fact, depletion of NK cells restored outgrowth of B16F10/M1KO cells, to similar extent as the growth of B16F10/PX458 cells (Fig 6B).

### Analysis of CRISPR/Cas9 induced mutations in treated EO-771 and B16F10 derived clones

Finally, we analyzed the CRISPR/Cas9 induced mutations among the selected β_2_m and IA beta chain KO clones. Therefore, the Cas9 target region within exon 1 of the β_2_m gene was amplified by PCR and the resulting PCR products were cloned and sequenced. Within B16F10/M1KO four different mutations were observed, occurring at the vicinity of the putative Cas9 cutting site within the crRNA-encoding target sequence (S3 Table, upper part). Insertions of a single nucleotide (C) or of di-nucleotides (TC) were found adjacent to position 58 in exon 1. Moreover, loss of a single nucleotide (C) at position 56, as well as deletion of a stretch of four nucleotides (GCTC) could be identified within the same clone. All insertions or deletions caused a frameshift in exon 1, thereby disrupting the reading frame. In contrast, no sequence changes were observed within the exon 1 of the β_2_m gene in the parental cell line and in control clone B16F10/PX458.

Analyses of the CRISPR/Cas9-induced sequence changes in the clone EO-NY/M1KO revealed a deletion of one nucleotide (G) at position 59, as well as loss of a hexanucleotide (TGGCTC) located at positions 53–58, and a deletion of a di-nucleotide (GC) at position 59–60 (S3 Table, lower part). Again, all mutations observed occurred adjacent to the predicted cutting site of the Cas9 nuclease. Whereas the deletions of the mono- and di-nucleotides resulted in frameshifts, deletion of the hexa-nucleotide TGGCTC did not alter the reading frame, but disrupted the start codon of exon 1. No sequence alterations were found in parental EO-NY cells and in EO-NY/PX458 control transfectants.

## Discussion

Seeking straight forward strategies for the generation of murine MHC deficient tumor cell lines we focused on the CRISPR/Cas9 based technology, as this technique was suggested to outperform classical strategies of targeted genome editing based on ZFPs or TALENs in frequency and precision [21]. In fact, usage of the construct PX458_mβ_2_m guide #1 was highly efficient, resulting in approximately 80% MHC class I negative B16F10 cells and EO-NY cells, respectively upon transfection, thereby exceeding mutation frequencies described for CRISPR/Cas9 induced genome editing in other murine cell lines [22].

Consecutive flow cytometric analysis of transfected cells revealed that PX458_mβ_2_m guide #1 mediated mutations occurred within three days after transfection, suggesting that intracellular expression of MHC class I heavy chains in the absence of β_2_m resulted in formation of instable heavy chain:peptide complexes that became degraded within the cytosol [23]. An exception represents the H2-D^b^ heavy chain, which can be stably expressed on the cell surface as single heavy chain, independent from associated β_2_m [24]. In our study, surface expression of free D^b^ heavy chains on the β_2_m KO transfectant clones can be excluded, because these would have been detected by monoclonal antibody (B22.249) which can recognize the conformation of free D^b^ heavy chains on the cell surface [25].

Our functional *in vitro* analyses clearly showed that the selected β_2_m KO clones of both tumor cell lines had lost susceptibility to CTL recognition, but became prone to recognition by NK cells. This result was verified in CTL assays including further β_2_m KO clones yielding almost identical results, thus demonstrating minimal clonal variation, at least with respect the susceptibility of individual KO clones to CTL recognition (not shown). The *in vitro* data were confirmed *in vivo* by tumor growth experiments showing that depletion of NK cells caused outgrowth of β_2_m KO B16F10 tumor cells in C57BL/6 mice.

Although we did not observe any phenotypical changes among our transfectant clones established, we cannot rule out occurrence of further mutations induced by the sgRNA-Cas9 complex outside the primary target sequences. In fact, off-target mutations differing by multiple nucleotide positions in the crRNA sequence or displaying a different PAM sequence have been described [26]. Meanwhile, strategies have been described to minimize possible off-target effects, for example by improving CRISPR-Cas9 specificity, using truncated sgRNAs, thereby increasing the stringency of the interaction resulting in fewer cleavages at off-target sites [27]. We maximized specificity by selecting guide sequences with high on-target scores and at least 3 bp mismatches to any predicted off-target sequences. Other authors developed strategies enhancing the specificity of Cas9 nucleases by combinatorial mutagenesis of positively charged amino acid stretches within the non-target strand binding groove of the SpCas9 complex, thereby reducing off-target cleavage events while retaining on-target specificity [28]. Similarly, amino acid exchanges at DNA contact sites within the Cas9 nuclease resulted in so called high fidelity Cas9 variants with enhanced on-target activity, but reduced off-target cleavage events, compared to wtCas9 [29].

The knowledge of T cell epitopes that are presented by human MHC molecules, i. e. of human leukocyte antigen (HLA)-restricted epitopes, is of crucial clinical relevance, since such epitopes could be included in synthetic peptide vaccines for the induction of tumor directed T cell responses in patients [30] or as follow-up tools during immune monitoring [31]. HLA-transgenic (HLAtg) mice have turned out as a useful tool for the identification of novel human tumor antigen specific, HLA-restricted T cell epitopes [32–34]. However until recently, most HLAtg mouse strains have been expressing the transgenic HLA molecule in conjunction with endogenous murine MHC (H2) molecules, resulting in interfering H2- and HLA-restricted T cell responses, particularly after immunization with global antigen [35]. Meanwhile, transgenic mouse strains have been established that co-express human MHC I and MHC II molecules in the absence of endogenous H2 molecules, hence the entire T cell repertoire becomes HLA-restricted in these mice [36]. As a remaining drawback, however, these mice are unsuitable to be used in tumor transplantation experiments with tumor cell lines still expressing murine MHC molecules on their cell surface, since such molecules would be recognized as xenogenic by the immune system of the HLAtg mouse. In such cases, surface expression of murine MHC molecules on the tumor cell lines would have to be eliminated prior to transplantation. In fact, the MHC negative tumor cell lines presented here could solve this problem by serving as parental lines for the establishment of MHC compatible, transplantable tumor models in HLA-transgenic mouse strains lacking expression of endogenous H2 molecules. Moreover, our tumor cell lines established may offer the possibility to investigate tumor reactive T cell responses that function independently from MHC molecule surface expression by the tumor. As it has been postulated that melanoma cells can be eradicated directly by CD4^+^ T cells [37], it would be interesting to use our B16F10/M2KO cell line as a control in tumor rejection experiments. As consequence, indirect anti-tumor effects of tumor antigen specific CD4^+^ T cells [38] could be analyzed with this cell line.

Naturally occurring loss of MHC I surface expression on tumor cells resulting from mutations in the β_2_m gene has been described for various tumor entities [39]. Although such mutations promote immune escape and selection of MHC I loss variants *in vivo*, these tumor cells rescued from recognition by MHC I restricted CTLs might still become susceptible to NK cells, known to preferentially target tumor cells devoid of MHC I surface expression [40]. Thus, our MHC I negative tumor cell lines might serve as useful tools to investigate MHC I restricted CTL responses versus NK cell activities *in vivo* and *in vitro*.

Studies performed with human primary cell lines have described CRISPR/Cas9 mediated elimination of β_2_m expression in hematopoietic stem cells [41] and very recently knock out of MHC II expression in primary human endothelial cells and murine multiple myeloma cells [42] by CRISPR/Cas9 targeting the CIIA locus was reported [43]. Our study may add to these results as we present for the first time the CRISPR/Cas9 mediated generation of stable MHC knockout melanoma and breast cancer cell lines of murine origin.

## Supporting information

S1 Fig Vector chart of PX458 used for targeted genome editing in murine tumor cell lines B16F10 and EO-NY. Oligomers complementary to predicted target sites within the genome were cloned into the Bbs1 site.
(TIF)

S2 Fig Murine tumor cell lines transfected with guide #1 enoding PX458 vector show loss of H2-D^b^ surface expression. EO-NY cells (a) and B16F10 cells (b) that had been transfected with construct PX458/#1 (right) or with control vector PX458 (middle) were sorted three days after transfection and tested for H2-D^b^ surface expression 7 days later by flow cytometry using H2-D^b^-specific monoclonal antibody B22.249. As control, autofluorescence of untreated tumor cell lines was determined (left).
(PPTX)

S3 Fig Analysis of H2-D^b^ surface expression on EO-NY-derived transfectant clones. H2-D^b^ surface expression of clones derived from EO-NY cells transfected with empty vector (EO-NY/PX458) or with guide#1 encoding vector (EO-NY/#1) was analyzed by flow cytometry. Untreated EO-NY cells were used as positive control (D^b^-APC) and to determine background signal intensities (unstained, isotype ctrl.). MFI values are given in the column at the right; designations of clones are depicted within the histograms.
(PPTX)

S4 Fig Analysis of H2-D^b^ surface expression on B16F10-derived transfectant clones. H2-D^b^ surface expression of B16F10 derived clones transfected with empty vector (B16F10 + PX458) or with guide#1 encoding vector (B16F10/#1) was analyzed by flow cytometry. Untreated B16F10 cells were used as positive control (D^b^-APC) and to determine background signal intensities (unstained, isotype ctrl.). MFI values are given in the column at the right; designations of clones are depicted within the histograms.
(PPTX)

S5 Fig Analysis of IA^b^ surface expression on B16F10 derived transfectant clones. IA^b^ surface expression of individual B16F10 derived clones transfected with guide #4 encoding vector and of parental B16F10 cells after treatment with IFNγ and subsequent staining with APC-conjugated IA^b^-specific monoclonal ab. Untreated (B16F10 w_o) and unstained B16F10 cells served as background controls, whereas parental B16F10 cells treated with IFNγ (B16F10 + IFNγ) served as positive control. Designations of clones are depicted in the column at the right.
(PPTX)

S1 Table Nucleotide sequences of primers used for the generation of target specific sgRNAs. Numbers in the right column represent on-target scores according to the CRISPR Design Tool (https://crispr.mit.edu/).
(DOCX)

S2 Table Primers used to for mutation analysis at genomic target sites. (DOCX)

S3 Table crRNA sequences and sequence analysis of mutated clones. crRNA sequences of used gRNAs are underlined; start codon of β_2_m exon 1 is highlighted in yellow; predicted Cas9 cutting sites are highlighted in red; PAM sequence is highlighted in green. Insertions are shown in red letters, red dashes represent deletions. In total, 14 or 15 bacterial clones derived from the knockout clones B16F10-M1KO or EO-NY-M1KO, respectively, were sequenced. We identified four different mutations for B16F10-M1KO and three different mutations for EO-NY-M1KO. The parental cell line B16F10 has been shown to be near tetraploid. The karyotype of parental EO-771 cells is unknown, but our results indicate trisomy of chromosome 2.
(DOCX)
