# Correction: Generation of murine tumor cell lines deficient in MHC molecule surface expression using the CRISPR/Cas9 system

**DOI:** 10.1371/journal.pone.0209719

**Published:** 2018-12-19

**Authors:** Krishna Das, David Eisel, Clarissa Lenkl, Ashish Goyal, Sven Diederichs, Elke Dickes, Wolfram Osen, Stefan B. Eichmüller

There are two errors in S2 Table. Labeling of primer sequences for PCR amplification are incorrect. Primers given for IA^b^β-chain are specific for β_2_m and vice versa. Furthermore, there is a mistake in the sequence for the nested forward primer specific for β_2_m.

**S2 Table:** Primers used to for mutation analysis at genomic target sites.

IA^b^ β-chain	forward	5'-TTTGCTTTCTGAAGGGGGCA-3'
	reverse	5'-AGAATGGAGTCTCACTCTCTCTT-3'
β_2_m	nested forward	5'-TCTGAGTGGGATATTGTCAGC-3'
	nested reverse	5'-TTGCGCTCAGGGAGTCTA-3'
	forward	5'-AAGGGTTGAGTTCTGCCAGTT-3'
	reverse	5'-TCTCTCGACTTCGGTTGGAT-3'
