# Supplementary figures and images for: Generation of murine tumor cell lines deficient in MHC molecule surface expression using the CRISPR/Cas9 system
Source: PLoS One. 2017 Mar 16;12(3):e0174077. doi: 10.1371/journal.pone.0174077 (PMC5354463; doi:10.1371/journal.pone.0174077)

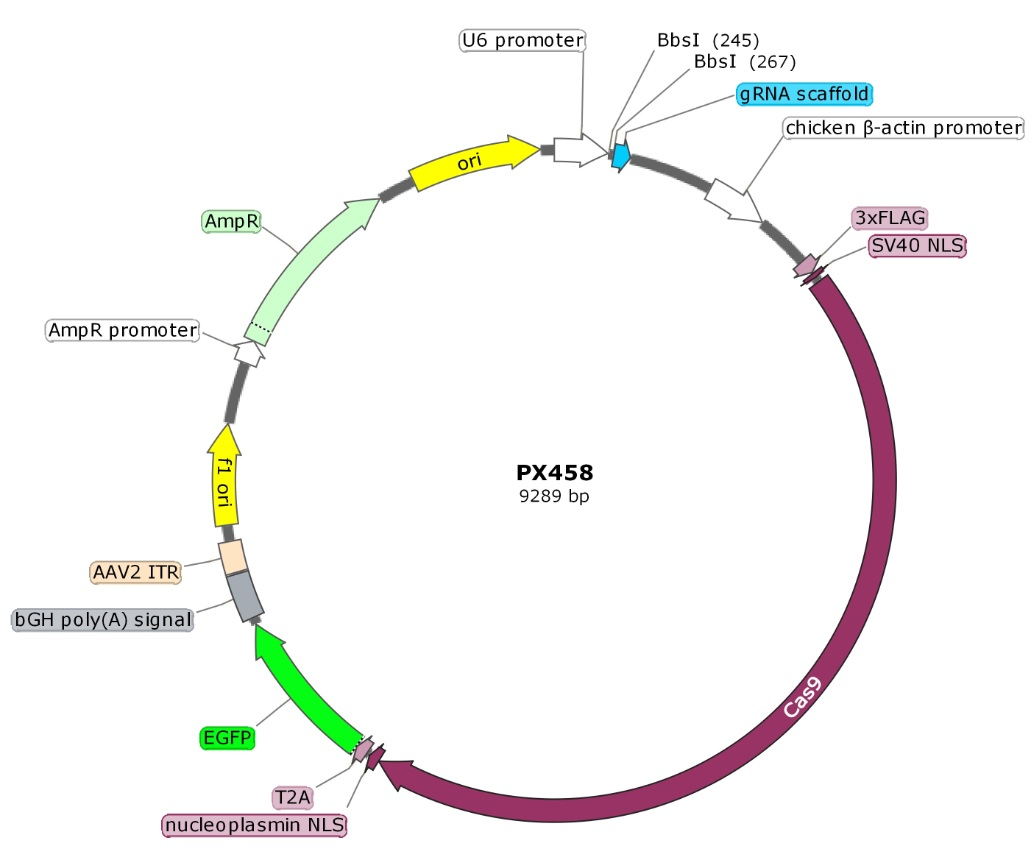

Supplement: S1 Fig — Oligomers complementary to predicted target sites within the genome were cloned into the Bbs1 site. (TIF) [file pone.0174077.s001.tif]

## Slide 1
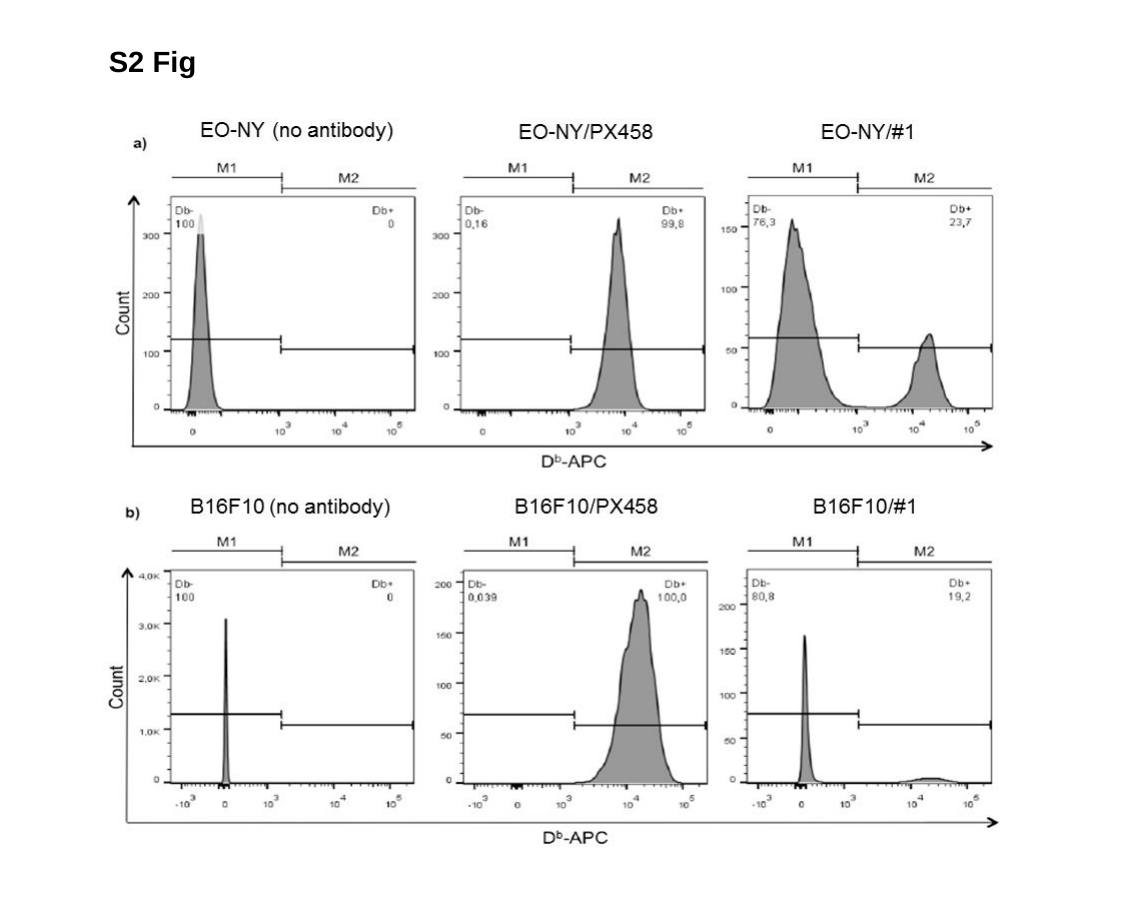

S2 Fig

Supplement: S2 Fig — EO-NY cells (a) and B16F10 cells (b) that had been transfected with construct PX458/#1 (right) or with control vector PX458 (middle) were sorted three days after transfection and tested for H2-Db surface expression 7 days later by flow cytometry using H2-Db-specific monoclonal antibody B22.249. As control, autofluorescence of untreated tumor cell lines was determined (left). (PPTX) [file pone.0174077.s002.pptx]

## Slide 1
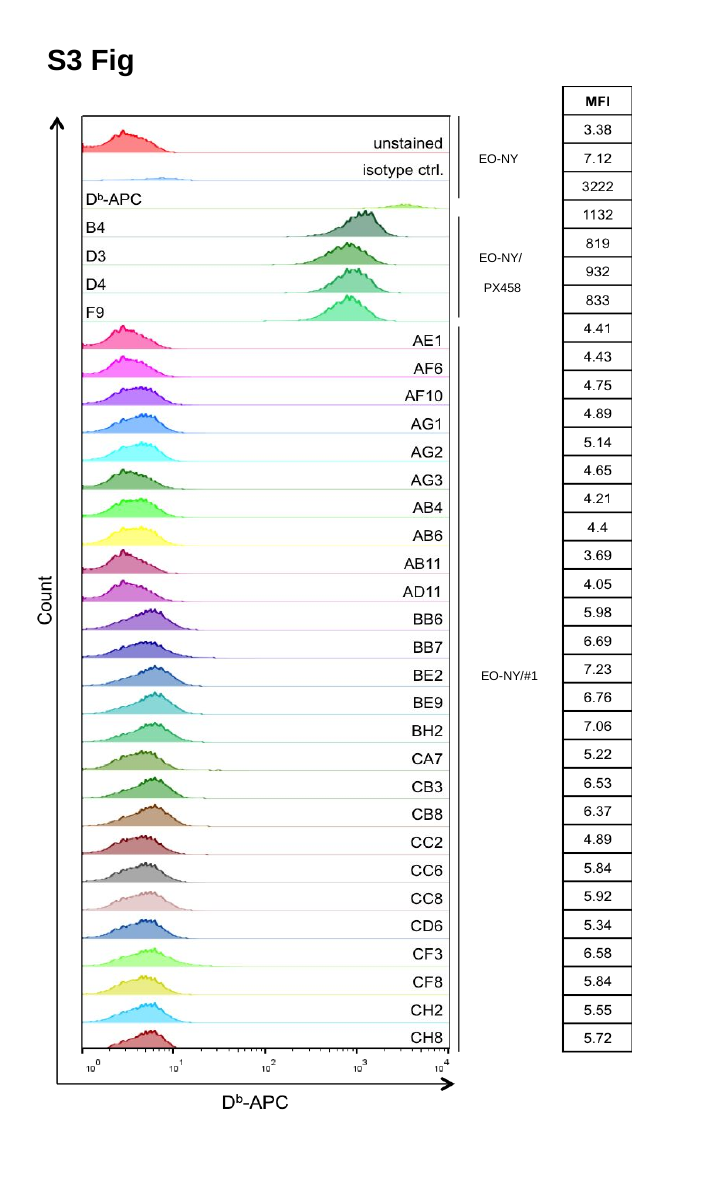

S3 Fig
EO-NY
EO-NY/ PX458
EO-NY/#1

Supplement: S3 Fig — H2-Db surface expression of clones derived from EO-NY cells transfected with empty vector (EO-NY/PX458) or with guide#1 encoding vector (EO-NY/#1) was analyzed by flow cytometry. Untreated EO-NY cells were used as positive control (Db-APC) and to determine background signal intensities (unstained, isotype ctrl.). MFI values are given in the column at the right; designations of clones are depicted within the histograms. (PPTX) [file pone.0174077.s003.pptx]

## Slide 1
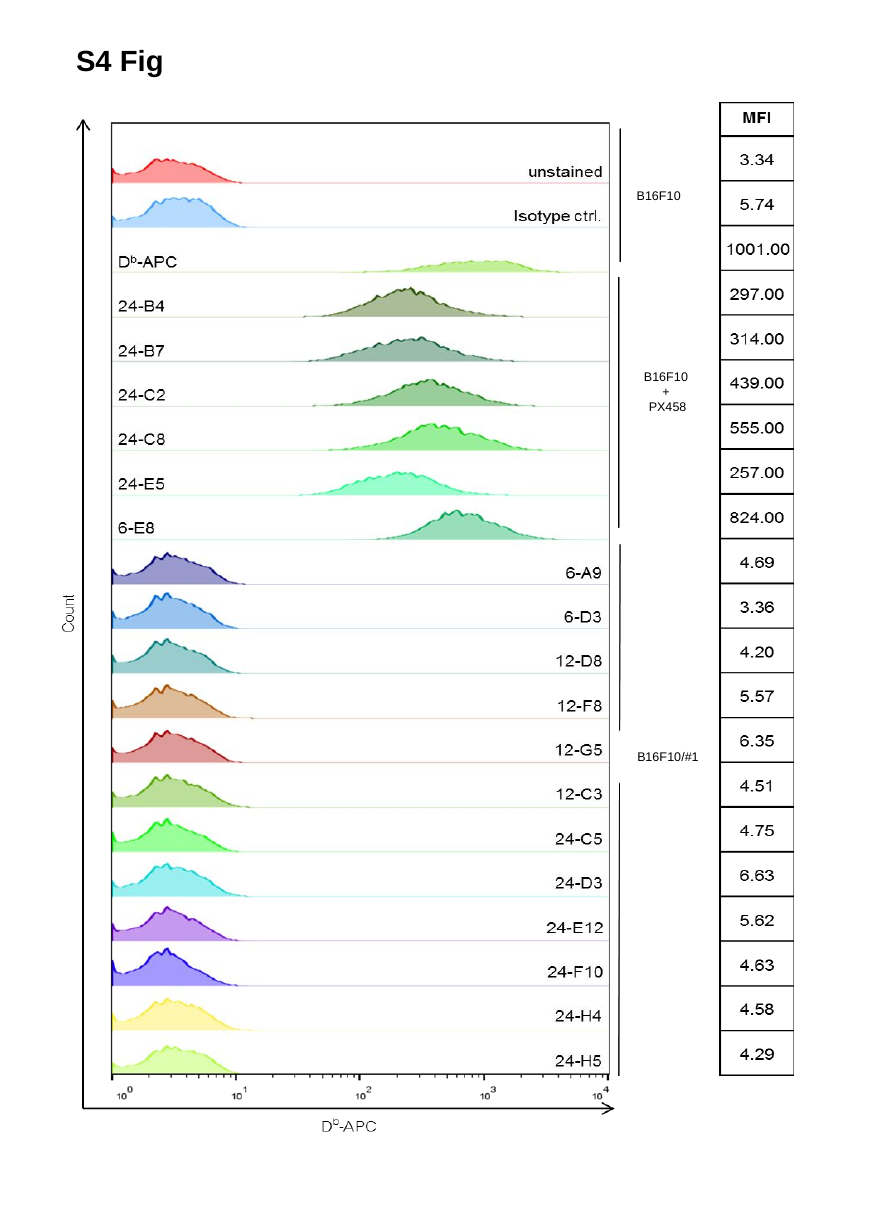

S4 Fig
B16F10
B16F10
+
PX458
B16F10/#1

Supplement: S4 Fig — H2-Db surface expression of B16F10 derived clones transfected with empty vector (B16F10 + PX458) or with guide#1 encoding vector (B16F10/#1) was analyzed by flow cytometry. Untreated B16F10 cells were used as positive control (Db-APC) and to determine background signal intensities (unstained, isotype ctrl.). MFI values are given in the column at the right; designations of clones are depicted within the histograms. (PPTX) [file pone.0174077.s004.pptx]

## Slide 1
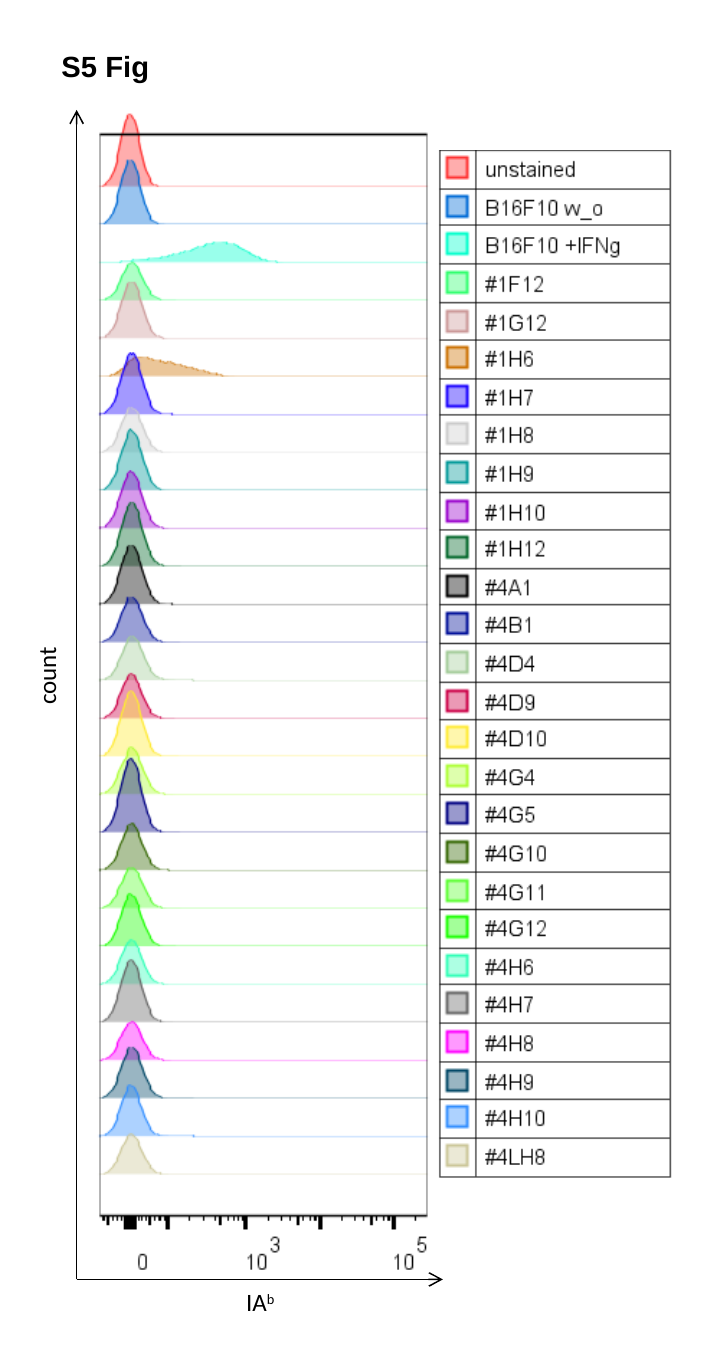

S5 Fig
count
IAb

Supplement: S5 Fig — IAb surface expression of individual B16F10 derived clones transfected with guide #4 encoding vector and of parental B16F10 cells after treatment with IFNγ and subsequent staining with APC-conjugated IAb-specific monoclonal ab. Untreated (B16F10 w_o) and unstained B16F10 cells served as background controls, whereas parental B16F10 cells treated with IFNγ (B16F10 + IFNγ) served as positive control. Designations of clones are depicted in the column at the right. (PPTX) [file pone.0174077.s005.pptx]
