# Supplementary material for: Generation of murine tumor cell lines deficient in MHC molecule surface expression using the CRISPR/Cas9 system
Source: PLoS One. 2017 Mar 16;12(3):e0174077. doi: 10.1371/journal.pone.0174077 (PMC5354463; doi:10.1371/journal.pone.0174077)
Supplement: S1 Table — Numbers in the right column represent on-target scores according to the CRISPR Design Tool (https://crispr.mit.edu/). (DOCX) [file pone.0174077.s006.docx]

| β_2_m | exon 1 guide #1 | crRNA sequence | 5'- GTCGTCAGCATGGCTCGCT-3' | 91 |
| --- | --- | --- | --- | --- |
|  |  | sense | 5'-CACCGTCGTCAGCATGGCTCGCT-3' |  |
|  |  | antisense | 5'-AAACAGCGAGCCATGCTGACGAC-3' |  |
|  | exon 1 guide #2 | crRNA sequence | 5'- GCATGGCTCGCTCGGTGACCC-3' | 87 |
|  |  | sense | 5'-CACCGCATGGCTCGCTCGGTGACCC-3' |  |
|  |  | antisense | 5'-AAACGGGTCACCGAGCGAGCCATGC-3' |  |
| IA^b^  β-chain | exon 1 guide #1 | crRNA sequence | 5'- GACTCCGAAAGTAAGTGCCG-3' | 95 |
|  |  | sense | 5'-CACCGACTCCGAAAGTAAGTGCCG-3' |  |
|  |  | antisense | 5'-AAACCGGCACTTACTTTCGGAGTC-3' |  |
|  | exon 1 guide #4 | crRNA sequence | 5'- GAGACTCCGAAAGTAAGTGC-3' | 80 |
|  |  | sense | 5'-CACCGAGACTCCGAAAGTAAGTGC-3' |  |
|  |  | antisense | 5'-AAACGCACTTACTTTCGGAGTCTC-3' |  |
