# Supplementary material for: Generation of murine tumor cell lines deficient in MHC molecule surface expression using the CRISPR/Cas9 system
Source: PLoS One. 2017 Mar 16;12(3):e0174077. doi: 10.1371/journal.pone.0174077 (PMC5354463; doi:10.1371/journal.pone.0174077)
Supplement: S2 Table — (DOCX) [file pone.0174077.s007.docx]

| β_2_m | forward | 5'-TTTGCTTTCTGAAGGGGGCA-3' |
| --- | --- | --- |
|  | reverse | 5'-AGAATGGAGTCTCACTCTCTCTT-3' |
| IA^b^  β-chain | nested forward | 5'-TCTGAGTGGATATTGTCAGC-3' |
|  | nested reverse | 5'-TTGCGCTCAGGGAGTCTA-3' |
|  | forward | 5'-AAGGGTTGAGTTCTGCCAGTT-3' |
|  | reverse | 5'-TCTCTCGACTTCGGTTGGAT-3' |
