# Supplementary material for: Generation of murine tumor cell lines deficient in MHC molecule surface expression using the CRISPR/Cas9 system
Source: PLoS One. 2017 Mar 16;12(3):e0174077. doi: 10.1371/journal.pone.0174077 (PMC5354463; doi:10.1371/journal.pone.0174077)
Supplement: S3 Table — crRNA sequences of used gRNAs are underlined; start codon of β2m exon 1 is highlighted in yellow; predicted Cas9 cutting sites are highlighted in red; PAM sequence is highlighted in green. Insertions are shown in red letters, red dashes represent deletions. In total, 14 or 15 bacterial clones derived from the knockout clones B16F10-M1KO or EO-NY-M1KO, respectively, were sequenced. We identified four different mutations for B16F10-M1KO and three different mutations for EO-NY-M1KO. The parental cell line B16F10 has been shown to be near tetraploid. The karyotype of parental EO-771 cells is unknown, but our results indicate trisomy of chromosome 2. (DOCX) [file pone.0174077.s008.docx]

**S3 Table**

| **Cell Line** | **Mutation** | **Sequence** | **Observed Frequency** |
| --- | --- | --- | --- |
| Murine β_2_m exon 1 (partial) |  | 5’-AGTCGTCAGC**ATG**GCTC\| GCT**CGG**-3’ |  |
|  |  |  |  |
| B16F10 |  | 5’-AGTCGTCAGC**ATG**GCTC\| GCT**CGG** | 100% |
| B16F10+PX458 |  | 5’-AGTCGTCAGC**ATG**GCTC\| GCT**CGG** | 100% |
| B16F10-M1KO | mut1 | 5’-AGTCGTCAGC**ATG**GCTC\| CGCT**CGG** | 29% (4/14) |
|  | mut2 | 5’-AGTCGTCAGC**ATG**GCTC\| TCGCT**CGG** | 21% (3/14) |
|  | mut3 | 5’-AGTCGTCAGC**ATG**G-TC\| GCT**CGG** | 29% (4/14) |
|  | mut4 | 5’-AGTCGTCAGC**ATG**GCTC\| ----**GG** | 21% (3/14) |

| Murine β_2_m exon 1 (partial) |  | 5’-AGTCGTCAGC**ATG**GCTC\| GCT**CGG**-3’ |  |
| --- | --- | --- | --- |
|  |  |  |  |
| EO-NY |  | 5’-AGTCGTCAGC**ATG**GCTC\| GCT**CGG** | 100% |
| EO-NY+PX458 |  | 5’-AGTCGTCAGC**ATG**GCTC\| GCT**CGG** | 100% |
| EO-NY-M1KO | mut1 | 5’-AGTCGTCAGC**ATG**GCTC\| -CT**CGG** | 40% (6/15) |
|  | mut2 | 5’-AGTCGTCAGC**A**------\| GCT**CGG** | 33% (5/15) |
|  | mut3 | 5’-AGTCGTCAGC**ATG**GCTC\| --T**CGG** | 27% (4/15) |
